# Clinical whole‐exome sequencing results impact medical management

**DOI:** 10.1002/mgg3.484

**Published:** 2018-10-14

**Authors:** Nancy Niguidula, Christina Alamillo, Layla Shahmirzadi Mowlavi, Zöe Powis, Julie S. Cohen, Kelly D. Farwell Hagman

**Affiliations:** ^1^ Ambry Genetics Aliso Viejo California; ^2^ Division of Neurogenetics and Hugo W. Moser Research Institute at Kennedy Krieger Institute Baltimore Maryland

**Keywords:** clinical utility, diagnostic exome sequencing, diagnostic odyssey, medical management, whole‐exome sequencing

## Abstract

**Background:**

Clinical diagnostic whole‐exome sequencing (WES) is a powerful tool for patients with undiagnosed genetic disorders. To demonstrate the clinical utility, we surveyed healthcare providers (HCP) about changes in medical management and treatment, diagnostic testing, reproductive planning, and use of educational services subsequent to WES testing.

**Methods:**

For a period of 18 months, an 18‐question survey was sent to HCPs attached to the WES reports. We analyzed the molecular diagnosis, patient clinical features, and the medical management changes reported in the returned surveys.

**Results:**

A total of 62 (2.2% of 2,876) surveys were returned, consisting of 37.1% patients with a positive or likely positive pathogenic alteration, 51.6% negative results, 9.7% uncertain findings, and 1 patient (1.6%) with a novel candidate finding. Overall, 100% of the HCPs of patients with positive or likely positive WES results (*n* = 23) and HCPs of patients with uncertain WES results (*n* = 6) responded positively to one of the 18 queries. Of note, 37.5% of the HCPs of patients with negative WES results (*n* = 32) responded positively to at least one query.

**Conclusion:**

Overall, these data clearly demonstrate the clinical utility of WES by demonstrating the impact on medical management irrespective of the exome result.

## 1 INTRODUCTION

For patients with genetic diseases, reaching a diagnosis brings an end to the expensive, time‐consuming, and potentially invasive diagnostic odyssey that burdens patients, families, and the healthcare system. Clinical whole‐exome sequencing (WES) is an effective diagnostic tool to pinpoint rare genetic alterations in an unbiased and efficient way for patients who have failed to receive a diagnosis despite extensive testing and workup. Several studies have shown that WES provides a diagnostic rate ranging from 25% to 40%, which is two to three times higher than traditional genetic testing methods.(Farwell et al., 2015; Iglesias et al., 2014; Lee et al., 2014; Retterer et al., 2016; Srivastava et al., 2014; Yang et al., 2013, 2014, 2013, 2014) Recent publications have also demonstrated that if WES were used as a first‐tier test, there would be a substantial reduced time to diagnosis and it is only a half to one‐quarter the cost of traditional testing.(Joshi et al., 2016; Monroe et al., 2016; Neveling et al., 2013; Soden et al., 2014; Stark et al., 2017; Tan et al., 2017; Vissers et al., 2017).

An early and accurate molecular diagnosis can lead to optimal care and dramatic prognostic improvements for patients and their families.(Fan et al., 2014; Fogel et al., 2014; Hanchard et al., 2013; Iglesias et al., 2014; Sawyer et al., 2016; Srivastava et al., 2014; Worthey et al., 2011) The clinical utility of WES has been measured by evaluating medical charts and HCP surveys to demonstrate the impact WES has on medical management and treatment strategies.(Hu et al., 2017; Iglesias et al., 2014; Meng et al., 2017; Soden et al., 2014; Srivastava et al., 2014; Tan et al., 2017; Valencia et al., 2015) The clinical factors measured include changes to family planning and testing, new specialist referrals, changes in patient care, medication or dietary changes, further diagnostic testing, enhanced surveillance, and prognosis expectations. HCPs typically order WES for a mix of phenotypes making follow‐up difficult across several disciplines and individual disease cohorts generally suffer from small sample sizes. Despite these limitations, clinical utility studies of pediatric neurodevelopmental disorders (NDDs), multiple congenital anomalies, and critically ill infants have reported a 26%–64% change in medical management for patients with positive WES results.(Hu et al., 2017; Iglesias et al., 2014; Meng et al., 2017; Soden et al., 2014; Tan et al., 2017; Valencia et al., 2015).

In this study, we evaluated the clinical utility of WES by surveying HCPs about changes in clinical management subsequent to receiving their patient's WES test results. The survey gathered information from the ordering HCP about changes in medication and treatment plans, prognosis and risk assessment, reproductive planning, and educational services subsequent to WES testing.

## 2 MATERIALS AND METHODS

### 2.1 Ethical compliance

Solutions Institutional Review Board approved this study by expedited review. Retrospective data analysis of anonymized data exempted the study from the requirement to receive consent from patients.

### 2.2 Clinical samples

The study gathered information from HCPs who had referred their patients for WES via a post‐test survey (Supporting information Figure S1). A total of 2,876 surveys were sent out between 27 May 2015 and 16 December 2016 and were attached to the end of the WES report. The only inclusion criterion was that the patient underwent WES testing at one commercial testing laboratory during this time frame. If a patient opted to obtain secondary findings in addition to the primary results from the WES test, the HCP was asked to answer the same set of questions specifically in regard to the patient's secondary finding results. The responses from the survey were linked to the patient's demographic information and WES results to account for patient indications, clinical features, and results. We offered a minimal incentive to the HCP to complete the survey; HCPs who completed a survey were entered into a drawing to win a $100 gift card to Amazon.com. Once the survey was closed and all data were collected, patient identifiers were removed for the study. The clinical histories provided by the referring HCP for each patient were carefully summarized and tabulated by a genetic counselor. The following data points were included age of the proband at the date of sample receipt, gender, consanguinity, previous clinical and/or differential diagnoses, a short clinical synopsis, family history, and an 18‐category organ system(s) involvement categorization. The genetic counselor also determined whether the proband had intellectual disability (ID), a positive brain magnetic resonance imaging (MRI) result, multiple congenital anomalies, seizures/epilepsy, ataxia, autism spectrum disorder, or psychiatric disease.

### 2.3 Exome sequencing, analysis, and reporting

Exome library preparation, sequencing, bioinformatics, and analysis were performed as previously described.(Farwell et al., 2015; Farwell Hagman et al., 2016) WES reports with findings in clinically characterized genes were classified into four categories based on the combined assessment of the pathogenicity of the variant along with the level of clinical correlation between the proband's phenotype with gestalt of symptoms typically associated with the reported gene(s): (a) positive: relevant alteration(s) detected; (b) likely positive: relevant alteration(s) detected; (c) uncertain: alteration(s) of uncertain clinical relevance detected; and (d) negative: no relevant alterations detected. If either the phenotypic overlap or the deleterious nature of the alterations was classified as uncertain, then the overall WES result category was uncertain. WES reports with findings in novel candidate genes, which are always reported as uncertain findings, were classified into two categories: (a) candidate or (b) suspected candidate, as previously described.(Farwell Hagman et al., 2016).

## 3 RESULTS

### 3.1 Demographics and clinical indications

A total of 2,876 surveys were requested and 62 (2.2%) completed surveys were returned to the clinical laboratory. The patient demographics included 28 (45.2%) female patients and 34 (54.8%) male patients, with a median age of 5.5 years at the time of sample submission (Table 1). The majority of patients were under age 5 years (48.5%), and the remaining patients were between age 6–18 years (33.8%), age 21–82 years (17.7%), and prenatal (3.2%). The indications for WES testing included a wide range of clinical phenotypes and are representative of WES ordering patterns in general. Patients with non‐specific and complex NDD were the most prevalent (33.9%, 21/62) indication. Multiple congenital anomalies (8.1%), cancer susceptibility (6.5%), movement disorders (4.8%), and cardiovascular symptoms (4.8%) were also reported phenotypes of the patients included in the study cohort.

**Table 1:** Demographics

	*N*	%
Age		
Prenatal	2	3.2
Age <5 year	29	45.3
Age 6–18 year	22	33.8
Adults	11	17.7
Gender		
Male	34	54.8
Female	28	45.2
Primary reason for referral		
Neurodevelopmental	21	33.9
Multiple congenital anomalies	5	8.1
Intellectual disability	5	8.1
Seizure	5	8.1
Cancer susceptibility	4	6.5
Brain abnormalities	4	6.5
Autism	3	4.8
Movement disorder	3	4.8
Cardiovascular	3	4.8
Neuromuscular	2	3.2
Skeletal	2	3.2
Ataxia/spasticity	1	1.6
Hematologic	1	1.6
Immune	1	1.6
Undergrowth/failure to thrive	1	1.6
Mixed phenotype: cancer, multiple congenital disorder	1	1.6
Primary results		
Positive	23	37.1
Uncertain	6	9.7
Negative	32	51.6
Novel	1	1.6
Secondary findings		
ACMG negative	53	85.5
ACMG positive	2	3.2
Not tested	7	11.3

### 3.2 Exome sequencing diagnoses

Of the 62 HCPs that responded to the survey, 37.1% (23) were from patients with a positive or likely positive pathogenic alteration, 51.6% (32) had no relevant gene findings, 9.7% (6) had uncertain findings, and 1 patient (1.6%) had a novel candidate finding (Table 1). These detection rates are representative of WES in general and are not statistically different than those we have previously reported (Farwell et al., 2015) (chi‐square 4 × 2 contingency table *p* = 0.206509). Exome sequencing identified 26 unique variants in the 23 patients with positive or likely positive results. The majority of the patients (60.9%) presented with atypical and overlapping neurodevelopmental or neuromuscular disorder symptoms. Of the 26 pathogenic variants identified, six were inherited from a parent, 16 variants were de novo, and four were likely de novo alterations but could not be determined because the father was not tested. One patient had two pathogenic alterations in two different genes (Table 2).

**Table 2:** Positive and uncertain cases

Age (years)	Primary reason for referral	Primary results	Gene	Molecular diagnosis
prenatal	Cardiovascular	Positive	*AARS2* (OMIM#612035; GenBank RefSeq NG_031952.1)	Combined oxidative phosphorylation deficiency 8
3	Intellectual disability	Positive	*FOXP1* (OMIM#605515; GenBank RefSeq NG_028243.1)	Intellectual disability with language impairment and with or without autistic features
3	Intellectual disability	Positive	*SYNGAP1* (OMIM#603384; GenBank RefSeq NG_016137.2)	Mental retardation, autosomal dominant 5
2	Multiple congenital anomalies	Positive	*FBN1* (OMIM#134797; GenBank RefSeq NG_008805.2)	Type 1 Fibrillinopathy
10	Multiple congenital anomalies	Positive	*KAT6B* (OMIM#605880; GenBank RefSeq NG_032048.1)	Genitopatellar/Say‐Barber‐Biesecker‐Young‐Simpson syndrome
2	Multiple congenital anomalies	Partial positive	*ACTG2* (OMIM#102545; GenBank RefSeq NG_034140.1)	Visceral myopathy
2	Neurodevelopmental	Positive	*SLC16A2* (OMIM#300095; GenBank RefSeq NG_011641.1)	Allan‐Herndon‐Dudley syndrome
2	Neurodevelopmental	Positive	*TCF4* (OMIM#602272; GenBank RefSeq NG_011716.2)	Pitt‐Hopkins syndrome
8	Neurodevelopmental	Likely positive	*EP300* (OMIM#602700; GenBank RefSeq NG_009817.1)	Rubinstein‐Taybi syndrome
2	Neurodevelopmental	Likely positive	*DDX3X* (OMIM#300160; GenBank RefSeq NG_012830.2)	X‐linked intellectual disability 102
		Positive	*RAI1* (OMIM#607642; GenBank RefSeq NG_007101.2)	Smith‐Magenis syndrome
1	Neurodevelopmental	Positive	*MECP2* (OMIM#300005; GenBank RefSeq NG_007107.2)	Encephalopathy, neonatal severe
4	Neurodevelopmental	Positive	*KDM5C* (OMIM#314690; GenBank RefSeq NG_008085.2) *MSH6* [Link] (OMIM#600678; GenBank RefSeq NG_007111.1)	X‐linked Claes‐Jensen type syndromic mental retardation, Lynch syndrome
3	Neurodevelopmental	Positive	*SLC2A1* (OMIM#138140; GenBank RefSeq NG_008232.1)	Stomatin‐deficient cryohydrocytosis with neurologic defects
21	Neurodevelopmental	Positive	*FOXP1* (OMIM#605515; GenBank RefSeq NG_028243.1)	Intellectual disability with language impairment and with or without autistic features
14	Neurodevelopmental	Positive	*ANKRD11* (OMIM#611192; GenBank RefSeq NG_032003.1)	KBG syndrome
2	Neurodevelopmental	Positive	*SCN8A* (OMIM#600702; GenBank RefSeq NG_021180.2)	Epileptic encephalopathy, early infantile, 13
1	Neurodevelopmental	Positive	*GABBR2* (OMIM#607340; GenBank RefSeq NG_016426.1)	GABBR2‐related neurodevelopmental disorder
7	Neurodevelopmental	Positive	*UBE3A* (OMIM#601623; GenBank RefSeq NG_009268.1)	Angelman syndrome
17	Neurodevelopmental	Positive	*PMM2* (OMIM#601785; GenBank RefSeq NG_009209.1)	Congenital disorder of glycosylation, Type IA
1 month.	Neuromuscular	Likely positive	*SOX10* (OMIM#602229; GenBank RefSeq NG_007948.1)	*SOX10*‐related peripheral demyelinating neuropathy
4	Neuromuscular	Positive	*TPM3* (OMIM#191030; GenBank RefSeq NG_008621.1)	*TPM3*‐related myopathy
4	Seizures	Likely positive	*MEF2C* (OMIM#600662; GenBank RefSeq NG_023427.1)	Mental retardation, stereotypic movements, epilepsy, and/or cerebral malformations
2	Cancer susceptibility, Multiple congenital disorders	Likely positive	*SMARCE1* (OMIM#603111; GenBank RefSeq NG_032163.1)	Coffin‐Sirissyndrome
		Expanded positive	*MSH6* (OMIM#600678; GenBank RefSeq NG_007111.1)	Increased risk of Lynch syndrome
4	Neurodevelopmental	Novel	*ZBTB18* (OMIM#608433; GenBank RefSeq NG_033841.1)	*ZBTB18*‐related intellectual disability with hypogenesis of the corpus callosum
5	Autism	Uncertain	*IQSEC2* (OMIM#300522; GenBank RefSeq NG_021296.2)	
1 month	Cardiovascular	Uncertain	*HCN4* (OMIM#605206; GenBank RefSeq NG_009063.1)	
23	Movement disorder	Uncertain	*TPK1* (OMIM#606370; GenBank RefSeq NG_032112.1)	
11	Neurodevelopmental	Uncertain	*SCN8A* (OMIM#600702; GenBank RefSeq NG_021180.2)	
8	Seizures	Uncertain	*DCX* (OMIM#300121; GenBank RefSeq NG_011750.1)	
11	Seizures	Uncertain	*SETBP1* (OMIM#611060;GenBank RefSeq NG_027527.2)	
7	Undergrowth	Positive[Link]	*CACNA1S* (OMIM#114208; GenBank RefSeq NG_009816.1)	Increased risk of malignant hyperthermia

Secondary finding.

### 3.3 Effect of WES on clinical management

**Figure 1:**
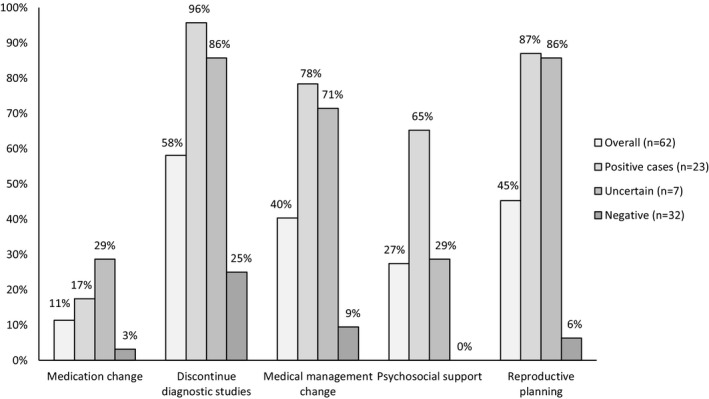
Medical management changes based on WES results. The survey results of the five categories of medical management stratified by the exome sequencing result. The percentage is based on the number of cases that responded positively to one of the queries within a specific category

To demonstrate the clinical utility of WES, we surveyed HCPs on 18 components of medical management, reproductive planning, and educational services after receiving a WES report. Overall, irrespective of WES results, 67.7% (42/62) of the HCPs responded positively to at least one of the 18 queries. When stratified by WES results, 100% of the HCPs of patients with positive or likely positive WES results (*n* = 23) and 100% of HCPs of patients with uncertain WES results (*n* = 7) responded positively to one of the 18 queries. Notably, 37.5% (12/32) of the HCPs of patients with negative WES results also responded positively to at least one query. To better evaluate the results, the survey was divided into five categories: (a) medication changes, (b) discontinuation of diagnostic testing, (c) medical management changes, (d) availability of psychosocial services, and (e) reproductive planning changes. Each of the five categories had two to five queries each (Supporting information Figure S1), and the results are summarized in Figure 1.

To demonstrate the impact WES specifically had on medical management, we grouped together the survey results from categories 1–3 (medication changes, discontinuation of further diagnostic studies, and medical management changes). Irrespective of the exome result, 64.5% (40/62) of the HCPs responded positively to a query within one of those three categories. When stratifying by WES result, the medical management changes for the patients with positive WES results, negative WES results, and uncertain WES results were 100%, 100%, and 31.3%, respectively.

### 3.4 Medication changes

Based on the surveys from HCPs of patients who received a positive WES result, 17.4% reported a change in medication (Table 3). The HCP for a patient with a dual diagnosis (X‐linked ID [*OMIM#300160]* and Smith‐Magenis syndrome [*OMIM#607642]*) responded positively to all three queries: new medication, discontinued unnecessary medication, and discontinued medication due to adverse effects. A patient with multiple congenital anomalies and a positive WES result qualified for a clinical trial and new treatments. One patient with a positive finding was prescribed a new ketogenic diet to reduce seizures based on the diagnosis of neurologic defects with stomatin‐deficient cryohydrocytosis (*OMIM#138140*). Interestingly, one patient with a negative result (3.1%) also had a medication change. The patient's indication for testing was suspected inherited skeletal syndrome and the negative result allowed the HCP to discontinue the patient's specialized ketogenic diet, presumably due to a ruled out differential diagnosis. Additionally, two HCPs of patients who received an uncertain WES result reported a new medication prescription.

**Table 3:** Survey results

	Positive patients	Uncertain patients[Link]	Negative patients
	*n* = 23	*n* = 7	*n* = 32
New medication	4 (17.4%)	2 (28.6%)	0%
Discontinue unnecessary medication	1 (4.3%)	0%	1 (3.1%)
Discontinue medication with adverse effects	1 (4.3%)	0%	0%
Discontinue diagnostic studies	15 (65.2%)	4 (57.1%)	4 (12.5%)
Discontinue genetic testing	21 (91.3%)	6 (85.7%)	8 (25%)
Referral to additional specialist	12 (52.2%)	2 (28.6%)	3 (9.4%)
Investigate additional manifestations	13 (56.5%)	2 (28.6%)	1 (3.1%)
Enhanced surveillance/prophylactic surgery	7 (30.4%)	3 (42.9%)	0%
Change prognosis expectations	14 (60.9%)	3 (42.9%)	1 (3.1%)
Availability of clinical trial	4 (17.4%)	0%	0%
Referral to support group	13 (56.5%)	1 (14.3%)	0%
Availability of additional education services	12 (52.2%)	2 (28.6%)	0%
Altered family financial planning	3 (13%)	0%	0%
Changed presumed inheritance pattern	9 (39.1%)	0%	1 (3.1%)
Established accurate recurrence risks	19 (82.6%)	2 (28.6%)	1 (3.1%)
Enabled reproductive planning options	11 (47.8%)	2 (28.6%)	1 (3.1%)
Gained option for carrier testing	4 (17.4%)	3 (42.9%)	0%
Enabled earlier diagnosis of relative	4 (17.4%)	2 (28.6%)	1 (3.1%)

Including one patient with a novel finding.

### 3.5 Discontinuation of diagnostic testing

Of the patients with positive WES results in this study, 65.2% of the HCPs reported a discontinuation of further diagnostic studies and 91.3% discontinued additional genetic tests (Table 3). In a patient with global developmental delay and spastic cerebral palsy who received an Allan‐Herndon‐Dudley syndrome (*OMIM#300095)* diagnosis, the ordering HCP commented that the exome test “ended the very aggressive diagnostic efforts” and the patient qualified for a clinical trial (Table 4). Among HCP surveys from patients with negative WES results, 12.5% discontinued further diagnostic studies and 25% discontinued genetic testing. A patient with NDD who received a negative WES result was able to narrow the diagnostic studies to additional enzyme activity and metabolic tests (Table 4). Nearly, all (85.7%) of the seven HCPs of patients with uncertain WES results discontinued genetic testing and 57.1% discontinued further diagnostic studies. The HCP of a patient with a complex epilepsy phenotype and an uncertain WES finding commented that the “test result ended their patient's diagnostic odyssey” which included NDD and neuromuscular assessments, five genetic tests, microarray, MRI, and metabolic panel testing (Table 4). The HCP of a patient with cardiovascular symptoms reported ordering additional deletion/duplication testing for one gene after receiving the uncertain WES result. The HCP of the patient with a novel finding responded that based on the WES result, they were able to discontinue any further diagnostic or genetic testing.

**Table 4:** Additional comments from referring providers

Age (years)	Results	Gene	Primary reason for referral	Provider comments
1 month	Uncertain	*HCN4*	Cardiovascular	“Parents were very relieved the results are likely de novo and no apparent ID associated with mutation found. Further testing for a deletion or duplication of the gene.”
2	Positive	*ACTG2*	Multiple congenital anomalies	“The patient is now a candidate for a transplant.”
1	Positive	*MECP2*	Neurodevelopmental	“Allowed for palliative care and end‐of‐life decisions to be discussed more thoroughly.”
2	Positive	*SLC16A2*	Neurodevelopmental	“This test stopped very aggressive diagnostic efforts, qualified him for a clinical trial, and there are other treatments that he is now receiving. The parents also benefited from knowing the mechanism of inheritance.”
1	Positive	*GABBR2*	Neurodevelopmental	“We are pleased to have additional information that will help us in the future for care.”
2	Positive	*SCN8A*	Neurodevelopmental	“Neurology may recommend medication changes.”
3	Positive	*SLC2A1*	Neurodevelopmental	“New dietary recommendations. We will start her on ketogenic diet to reduce/control seizures and the movement disorder.”
1 month	Likely positive	*SOX10*	Neuromuscular	“The test provided clarification of prognosis expectations and helped to make decisions regarding care and long term.”
11	Uncertain	*SETBP1*	Seizures	“This test result ended the patient's diagnostic odyssey. There is no longer a need for unnecessary tests and the family can finally move on.”
4	Negative	N/A	Autism	“The result affected the diagnostic direction.”
27	Negative	N/A	Cancer susceptibility	“We will now pursue research testing.”
17	Negative	N/A	Immune	“The results helped to suggest that the symptoms are more likely viral than genetic or hereditary.”
16	Negative	N/A	Intellectual disability	“The patient is now eligible to enroll in the CURE's Epilepsy Genetics Initiative.”
11	Negative	N/A	Neurodevelopmental	“The results help direct the ordering of additional tests to evaluate variants (e.g. enzyme assay for *MANBA* and transferrin testing for *COG1*‐CDG).”
41	Negative	N/A	Skeletal	“The report allowed for the discontinuation of specialized diet and enabled reassurance to a 1st degree relative concerned about a genetic disorder.”

Note

The *MANBA* gene codes for the beta‐mannosidase metabolizing enzyme.

COG1‐CDG; *COG1* gene‐related congenital disorders of glycosylation.

### 3.6 Medical management changes

Based on the surveys from the HCPs of the patients with a positive or likely positive WES result, 78.3% reported a change in medical management (Figure 1). More than half of the HCPs of patients who received a definitive molecular diagnosis were able to refer the patient to an additional specialist (52.2%), investigate additional manifestations (56.5%), and/or changed the patient's prognosis (60.9%). A third (30.4%) of the HCPs of patients with positive WES results enhanced surveillance or opted for prophylactic surgery, and 17.4% identified the patient's eligibility for a clinical trial (Table 3). The HCP of a patient who received a diagnosis of severe neonatal encephalopathy commented that the family was able to change their prognosis expectations since the exome result “allowed for palliative care and end‐of‐life decisions to be discussed more thoroughly” (Table 4). The HCP of a patient with multiple congenital anomalies who received a visceral myopathy diagnosis commented that the WES result affected the patient's eligibility as a transplant candidate. The HCP of the patient with a novel finding only responded to one medical management query which was a new referral to an additional specialist.

Of the seven patients with uncertain WES results, five (71.5%) of their HCPs reported a change to their medical management. Despite receiving an uncertain finding, the HCP of one patient reported that the patient underwent four of the five medical management changes. Two additional patients with uncertain WES results began an enhanced surveillance strategy, and another patient's family responded that the uncertain result prompted a change in prognosis expectations. Based on the surveys from HCP of patients with negative WES results, two patients had a change in medical management and three patients were referred to additional specialists. The HCP of a 3‐year‐old man with autistic symptoms commented that the negative WES result “affected the diagnostic direction” and referred the patient to an additional specialist for the investigation of other manifestations (Table 4). The HCP of a patient with an immune disorder and a negative WES result commented that the report “helped to suggest that the symptoms were more likely viral than of genetic origin”. A patient with a negative WES result, a 15‐year‐old patient with developmental delays, autism, gastrointestinal issues, and a history of seizures, did not have any of the survey queries answered, but the HCP commented that “the patient is now eligible to enroll in the CURE's Epilepsy Genetics Initiative.”

### 3.7 Availability of psychosocial services

In the psychosocial services component of the survey, 65.2% of the HCPs of patients with positive WES results responded positively to one of the three queries (Figure 1). Over half were referred to a support group (56.5%) and/or additional educational services (52.2%); however, only a few (13%) responded that their patient's families altered their financial planning (Table 3). None of the HCPs of patients with negative WES results reported the use of psychosocial services, while two of the seven HCPs of patients with uncertain results referred the patient to a support group and/or utilize educational services.

### 3.8 Reproductive planning changes

Irrespective of exome result, 45.2% of the cohort was able to initiate new reproductive planning options. Of the families of patients with positive WES results, the HCPs were able to establish accurate recurrence risks (82.6%), enable reproductive planning options (47.8%), and change the presumed inheritance pattern (39.1%). Additionally, 17.4% of the HCPs of patients with positive WES results provided the option for carrier testing of family members and 13% enabled the earlier diagnosis of an affected/asymptomatic relative (Table 3). The HCPs of six patients (85.7%) with uncertain WES results responded positively to one of the five reproductive planning queries. The HCP of a 7‐year‐old patient with seizures and an uncertain finding responded positively to four of the five reproductive planning questions. The HCP of an infant with cardiovascular anomalies and an uncertain exome result reported that reproductive planning options were now available for the family and that the “parents were relieved that it was a likely de novo variant and no variants were found associated to an ID” (Table 4). The HCPs of two patients (6.3%) with negative WES results responded positively to at least one of the reproductive planning queries. The HCP of a prenatal patient with negative WES results and multiple congenital disorders responded that the family was able to change the presumed inheritance pattern, establish recurrence risks, and enabled their reproductive planning options.

### 3.9 Secondary findings

The American College of Medical Genetics and Genomics (ACMG) published a list of 59 known pathogenic or expected pathogenic genes that should be reported as incidental or secondary findings when clinical laboratories perform exome sequencing, even when unrelated to the primary medical reason for testing.(Green et al., 2013; Kalia et al., [Link]) Additionally, our laboratory offers expanded secondary findings options from among ~1,200 genes.(Shahmirzadi et al., 2014) Of the 56 patients that opted for secondary findings results, two patients (3.6%) were identified with positive results: one result (1.8%) within one of the ACMG recommended secondary findings genes and one result (1.8%) within one of our databases expanded secondary findings genes (Tables 1 and 2). A 6.5 year old patient presenting with undergrowth or failure to thrive had a negative primary WES result; yet, a secondary finding was identified in *CACNA1S* (OMIM #114208; GenBank RefSeq NG_009816.1). The HCP responded in the survey that the primary result provided no significant changes in medical care, but the secondary findings enabled an earlier diagnosis of an affected/asymptomatic relative. The second patient was a 4.1 year old with a NDD whose primary result (*KDM5C;* OMIM #314690; GenBank RefSeq NG_008085.2) provided an X‐linked Claes‐Jensen syndrome diagnosis, and the secondary findings result indicated an increased risk of Lynch syndrome based on a pathogenic variant found in *MSH6* (OMIM #600678; GenBank RefSeq NG_007111.1). The HCP responded that the primary results led to the discontinuation of further diagnostic and genetic tests, while the secondary result enabled an early diagnosis of an affected/asymptomatic relative and enhanced their surveillance strategy. The HCP also responded that both results changed the presumed inheritance pattern and allowed the option for carrier testing in family members.

## 4 DISCUSSION

Of the surveys received, the majority of the patients had symptoms of complex NDDs, followed by patients with multiple congenital anomalies, ID, and seizures. The clinical characteristics of this cohort is similar to standard exome ordering patterns among patients referred to a commercial testing laboratory for WES with ID and/or developmental delay, positive brain MRI results, multiple congenital anomalies, and epilepsy (64%, 64%, 28%, and 24%, respectively; Farwell et al., 2015).

WES has proven to be an effective diagnostic tool with demonstrated clinical validity, high diagnostic yield, and reduced time to diagnosis. A retrospective review of 40 pediatric patients referred for WES showed that 48% had at least four genetic tests prior to WES, and several patients had more than 10 genetic tests.(Valencia et al., 2015) Several studies of pediatric NDDs or epilepsy patients have demonstrated a significant reduced time to diagnosis, with WES a diagnosis received in 3–5 months compared to 2–8 years with traditional methods.(Joshi et al., 2016; Soden et al., 2014; Tan et al., 2017; Vissers et al., 2017; Yavarna et al., 2015) Most importantly, two pediatric studies have shown that if WES were used as a first‐tier diagnostic test, 24%–64% of patients would have avoided invasive medical procedures.(Tan et al., 2017; Vissers et al., 2017) In this study, we surveyed the HCPs use of diagnostic procedures subsequent to WES testing. Overall, 95.7% of patients with positive WES results and 83.3% of patients with uncertain WES results were able to discontinue further diagnostic studies. While a negative result may be seen as somewhat uninformative, 25% of the HCPs of patients with negative WES results responded that they discontinued genetic testing specifically.

To demonstrate the impact WES specifically had on medical management, we grouped together the survey results from categories 1–3 (medication changes, discontinuation of further diagnostic studies, and medical management changes). Irrespective of the exome result, 64.5% (40/62) of the HCPs responded positively to a query within one of those three categories. One of the limitations of this study was that data were collected through December 2016, and clinical practices may have changed since then. Genes that were once considered uncertain may be pathogenic at this time and could impact HCP management decisions. While our results may be affected by survey bias based on the very low response rate (2%) and the HCPs who made changes in their patient's care may have been more motivated to complete and return the survey, our results are only slightly higher than results reported in similar publications. Previous studies of pediatric NDDs, multiple congenital anomalies, and critically ill infants have reported a 26%–52% change in medical management for patients with positive WES results.(Hu et al., 2017; Meng et al., 2017; Soden et al., 2014; Tan et al., 2017) Studies that evaluated the clinical utility of WES diagnoses in pediatric and young adult cancer patients have observed similar rates (46%–75%) of clinical utility.(Mody et al., 2015; Oberg et al., 2016) In our cohort, more than half of HCPs of patients with positive WES results reported a referral to an additional specialist, investigating additional manifestations, and a change in prognosis. Our cohort observed a higher percentage of medical management changes compared to a similar pediatric NDD study where 12.5% of patients with positive WES results underwent disease monitoring, 18.8% underwent further clinical workup, and 31.3% altered their prognosis.(Srivastava et al., 2014) In a WES study of 278 critically ill infants, the initiation of new specialist care was reported in 50.9%, the informed redirection of care was undertaken for 35.8%, and major procedures were ordered for 9.4% of patients with positive WES results (Meng et al., 2017).

In the medication category, HCPs of patients who received a positive WES results, uncertain result, and negative result reported a change in care (17.4%, 33.3%, and 3.1%, respectively). These results are similar to pediatric studies of ID and NDDs with 21.9%–22.2% of patients with positive WES results reporting a new drug or dietary treatment.(Soden et al., 2014; Srivastava et al., 2014) In the WES study of unrelated critically ill infants, 13.2% of patients with a definitive diagnosis altered their medication and/or dietary treatments (Meng et al., 2017). Based on the patients with positive WES results in our cohort, 82.6% were given accurate recurrence risks, 47.8% were given reproductive planning options, and 39.1% were informed of a change in the presumed inheritance pattern. The Srivastava et al. study of pediatric ND patients (*n* = 78) is the only study that queried changes in reproductive planning changes. Of the 84.4% (27/32) patients with a positive WES diagnosis, the results were essential for reproductive planning and an additional seven (21.9%) patients changed their presumed inheritance pattern (Srivastava et al., 2014).

While an uncertain or negative exome finding may seem uninformative, we are the first to demonstrate that these results have an impact on medical management. In this cohort, two patients with uncertain WES results started new medications and one patient with a negative WES result discontinued a specialized diet. Additionally, 66.7% of the patients with uncertain WES results had a change in clinical management and 9.4% of the patients with negative WES results were given a referral to an additional specialist. We also observed that patients with uncertain results were referred for psychosocial services, and the majority made reproductive planning changes. As noted in Table 4, the HCPs of six patients with negative WES results and two patients with uncertain WES results provided additional comments on the impact WES testing had on their patient's medical management.

In the past few years, there has been a surge of WES publications demonstrating a higher diagnostic yield, reduced time to diagnosis, clinical utility, and cost‐effectiveness compared to the standard diagnostic pathway. (Farwell et al., 2015; Iglesias et al., 2014; Joshi et al., 2016; Lee et al., 2014; Monroe et al., 2016; Neveling et al., 2013; Retterer et al., 2016; Soden et al., 2014; Srivastava et al., 2014; Stark et al., 2017; Tan et al., 2017; Vissers et al., 2017; Yang et al., 2013, 2014 ) Several studies have evaluated the cost‐effectiveness for complex pediatric neurologic patients and demonstrate a 24%–40% reduction in diagnostic costs when using WES as a first‐tier test compared to traditional diagnostic methods.(Joshi et al., 2016; Monroe et al., 2016; Neveling et al., 2013; Soden et al., 2014; Vissers et al., 2017) It is evident that the time and cost of uninformative tests in the traditional diagnostic pathway for complex NDDs and multiple congenital anomalies warrant WES as a first‐tier test. In this study, we demonstrate 100% clinical utility of WES based on HCP surveys of patients with positive or uncertain WES results among an unbiased cohort of patients referred for WES. Additionally, we are the first to demonstrate that even negative WES results have an impact on medical management. Collectively, the WES studies that have evaluated the impact WES has on medical management and patient outcome clearly demonstrates the clinical utility of this diagnostic tool.

## CONFLICT OF INTEREST

K.D.F.H., L.S.M., C.L.A., Z.P., and N.N. are employed by and receive a salary from Ambry Genetics. J.S.C. is a consultant for Invitae. Exome sequencing is among the commercially available tests at these laboratories.

## References

[mgg3484-bib-0001] Fan, Z. , Greenwood, R. , Felix, A. C. , Shiloh‐Malawsky, Y. , Tennison, M. , Roche, M. , … Evans, J. (2014). GCH1 heterozygous mutation identified by whole‐exome sequencing as a treatable condition in a patient presenting with progressive spastic paraplegia. Journal of Neurology, 261(3), 622–624. 10.1007/s00415-014-7265-3 24509643 PMC4000021

[mgg3484-bib-0002] Farwell Hagman, K. D. , Shinde, D. N. , Mroske, C. , Smith, E. , Radtke, K. , Shahmirzadi, L. , … Tang, S. (2016). Candidate‐gene criteria for clinical reporting: Diagnostic exome sequencing identifies altered candidate genes among 8% of patients with undiagnosed diseases. Genetics in Medicine, 19, 224–235. 10.1038/gim.2016.95 27513193 PMC5303763

[mgg3484-bib-0003] Farwell, K. D. , Shahmirzadi, L. , El‐Khechen, D. , Powis, Z. , Chao, E. C. , Tippin Davis, B. , … Tang, S. (2015). Enhanced utility of family‐centered diagnostic exome sequencing with inheritance model‐based analysis: Results from 500 unselected families with undiagnosed genetic conditions. Genetics in Medicine, 17(7), 578–586. 10.1038/gim.2014.154 25356970

[mgg3484-bib-0004] Fogel, B. L. , Lee, H. , Deignan, J. L. , Strom, S. P. , Kantarci, S. , Wang, X. , … Nelson, S. F. (2014). Exome sequencing in the clinical diagnosis of sporadic or familial cerebellar ataxia. JAMA Neurol, 71(10), 1237–1246. 10.1001/jamaneurol.2014.1944 25133958 PMC4324730

[mgg3484-bib-0005] Green, R. C. , Berg, J. S. , Grody, W. W. , Kalia, S. S. , Korf, B. R. , Martin, C. L. , Biesecker, … L. G. (2013). ACMG recommendations for reporting of incidental findings in clinical exome and genome sequencing. Genetics in Medicine, 15(7), 565–574. 10.1038/gim.2013.73 23788249 PMC3727274

[mgg3484-bib-0006] Hanchard, N. A. , Murdock, D. R. , Magoulas, P. L. , Bainbridge, M. , Muzny, D. , Wu, Y. , … Brown, C. W. (2013). Exploring the utility of whole‐exome sequencing as a diagnostic tool in a child with atypical episodic muscle weakness. Clinical Genetics, 83(5), 457–461. 10.1111/j.1399-0004.2012.01951.x 22901280 PMC3926310

[mgg3484-bib-0007] Hu, X. , Li, N. , Xu, Y. , Li, G. , Yu, T. , Yao, R. E. , … Shen, Y. (2017). Proband‐only medical exome sequencing as a cost‐effective first‐tier genetic diagnostic test for patients without prior molecular tests and clinical diagnosis in a developing country: The China experience. Genetics in Medicine, 20 (9), 1045–1053. 10.1038/gim.2017.195 29095814

[mgg3484-bib-0008] Iglesias, A. , Anyane‐Yeboa, K. , Wynn, J. , Wilson, A. , Truitt Cho, M. , Guzman, E. , Chung, … W. K. (2014). The usefulness of whole‐exome sequencing in routine clinical practice. Genetics in Medicine, 16, 922–931. 10.1038/gim.2014.58 24901346

[mgg3484-bib-0009] Joshi, C. , Kolbe, D. L. , Mansilla, M. A. , Mason, S. O. , Smith, R. J. , & Campbell, C. A. (2016). Reducing the cost of the diagnostic odyssey in early onset epileptic encephalopathies. BioMed Research International, 2016, 6421039 10.1155/2016/6421039 27243033 PMC4875968

[mgg3484-bib-0010] Kalia, S. S. , Adelman, K. , Bale, S. J. , Chung, W. K. , Eng, C. , Evans, J. P. , … Miller, D. T. (2017). Recommendations for reporting of secondary findings in clinical exome and genome sequencing, 2016 update (ACMG SF v2.0): a policy statement of the American College of Medical Genetics and Genomics. Genetics in Medicine, 19(2), 249–255. 10.1038/gim.2016.190 27854360

[mgg3484-bib-0011] Lee, H. , Deignan, J. L. , Dorrani, N. , Strom, S. P. , Kantarci, S. , Quintero‐Rivera, F. , … Nelson, S. F. (2014). Clinical exome sequencing for genetic identification of rare Mendelian disorders. JAMA, 312(18), 1880–1887. 10.1001/jama.2014.14604 25326637 PMC4278636

[mgg3484-bib-0012] Meng, L. , Pammi, M. , Saronwala, A. , Magoulas, P. , Ghazi, A. R. , Vetrini, F. , … Lalani, S. R. (2017). Use of exome sequencing for infants in intensive care units: Ascertainment of severe single‐gene disorders and effect on medical management. JAMA Pediatrics, 171(12), e173438 10.1001/jamapediatrics.2017.3438 28973083 PMC6359927

[mgg3484-bib-0013] Mody, R. J. , Wu, Y. M. , Lonigro, R. J. , Cao, X. , Roychowdhury, S. , Vats, P. , … Chinnaiyan, A. M. (2015). Integrative clinical sequencing in the management of refractory or relapsed cancer in youth. JAMA, 314(9), 913–925. 10.1001/jama.2015.10080 26325560 PMC4758114

[mgg3484-bib-0014] Monroe, G. R. , Frederix, G. W. , Savelberg, S. M. , de Vries, T. I. , Duran, K. J. , van der Smagt, J. J. , … van Haaften, G. (2016). Effectiveness of whole‐exome sequencing and costs of the traditional diagnostic trajectory in children with intellectual disability. Genetics in Medicine, 18(9), 949–956. 10.1038/gim.2015.200 26845106

[mgg3484-bib-0015] Neveling, K. , Feenstra, I. , Gilissen, C. , Hoefsloot, L. H. , Kamsteeg, E. J. , Mensenkamp, A. R. , … Nelen, M. R. (2013). A post‐hoc comparison of the utility of sanger sequencing and exome sequencing for the diagnosis of heterogeneous diseases. Human Mutation, 34(12), 1721–1726. 10.1002/humu.22450 24123792

[mgg3484-bib-0016] Oberg, J. A. , Glade Bender, J. L. , Sulis, M. L. , Pendrick, D. , Sireci, A. N. , Hsiao, S. J. , … Kung, A. L. (2016). Implementation of next generation sequencing into pediatric hematology‐oncology practice: Moving beyond actionable alterations. Genome Medicine, 8(1), 133 10.1186/s13073-016-0389-6 28007021 PMC5180407

[mgg3484-bib-0017] Retterer, K. , Juusola, J. , Cho, M. T. , Vitazka, P. , Millan, F. , Gibellini, F. , … Bale, S. (2016). Clinical application of whole‐exome sequencing across clinical indications. Genetics in Medicine, 18(7), 696–704. 10.1038/gim.2015.148 26633542

[mgg3484-bib-0018] Sawyer, S. L. , Hartley, T. , Dyment, D. A. , Beaulieu, C. L. , Schwartzentruber, J. , Smith, A. , … Boycott, K. M. (2016). Utility of whole‐exome sequencing for those near the end of the diagnostic odyssey: Time to address gaps in care. Clinical Genetics, 89(3), 275–284. 10.1111/cge.12654 26283276 PMC5053223

[mgg3484-bib-0019] Shahmirzadi, L. , Chao, E. C. , Palmaer, E. , Parra, M. C. , Tang, S. , & Gonzalez, K. D. (2014). Patient decisions for disclosure of secondary findings among the first 200 individuals undergoing clinical diagnostic exome sequencing. Genetics in Medicine, 16(5), 395–399. 10.1038/gim.2013.153 24113345 PMC4018499

[mgg3484-bib-0020] Soden, S. E. , Saunders, C. J. , Willig, L. K. , Farrow, E. G. , Smith, L. D. , Petrikin, J. E. , … Kingsmore, S. F. (2014). Effectiveness of exome and genome sequencing guided by acuity of illness for diagnosis of neurodevelopmental disorders. Science Translational Medicine, 6(265), 265ra168 10.1126/scitranslmed.3010076 PMC4286868 25473036

[mgg3484-bib-0021] Srivastava, S. , Cohen, J. S. , Vernon, H. , Baranano, K. , McClellan, R. , Jamal, L. , … Fatemi, A. (2014). Clinical whole exome sequencing in child neurology practice. Annals of Neurology, 76(4), 473–483. 10.1002/ana.24251 25131622

[mgg3484-bib-0022] Stark, Z. , Schofield, D. , Alam, K. , Wilson, W. , Mupfeki, N. , Macciocca, I. , … Gaff, C. (2017). Prospective comparison of the cost‐effectiveness of clinical whole‐exome sequencing with that of usual care overwhelmingly supports early use and reimbursement. Genetics in Medicine, 19(8), 867–874. 10.1038/gim.2016.221 28125081

[mgg3484-bib-0023] Tan, T. Y. , Dillon, O. J. , Stark, Z. , Schofield, D. , Alam, K. , Shrestha, R. , … White, S. M. (2017). Diagnostic impact and cost‐effectiveness of whole‐exome sequencing for ambulant children with suspected monogenic conditions. JAMA Pediatrics, 171(9), 855–862. 10.1001/jamapediatrics.2017.1755 28759686 PMC5710405

[mgg3484-bib-0024] Valencia, C. A. , Husami, A. , Holle, J. , Johnson, J. A. , Qian, Y. , Mathur, A. , … Zhang, K. (2015). Clinical impact and cost‐effectiveness of whole exome sequencing as a diagnostic tool: A pediatric center's experience. Frontiers in Pediatrics, 3, 67 10.3389/fped.2015.00067 26284228 PMC4522872

[mgg3484-bib-0025] Vissers, L. , van Nimwegen, K. J. M. , Schieving, J. H. , Kamsteeg, E. J. , Kleefstra, T. , Yntema, H. G. , … Willemsen, M. (2017). A clinical utility study of exome sequencing versus conventional genetic testing in pediatric neurology. Genetics in Medicine, 19(9), 1055–1063. 10.1038/gim.2017.1 28333917 PMC5589982

[mgg3484-bib-0026] Worthey, E. A. , Mayer, A. N. , Syverson, G. D. , Helbling, D. , Bonacci, B. B. , Decker, B. , … Dimmock, D. P. (2011). Making a definitive diagnosis: Successful clinical application of whole exome sequencing in a child with intractable inflammatory bowel disease. Genetics in Medicine, 13(3), 255–262. 10.1097/GIM.0b013e3182088158 21173700

[mgg3484-bib-0027] Yang, Y. , Muzny, D. M. , Reid, J. G. , Bainbridge, M. N. , Willis, A. , Ward, P. A. , … Eng, C. M. (2013). Clinical whole‐exome sequencing for the diagnosis of mendelian disorders. New England Journal of Medicine, 369(16), 1502–1511. 10.1056/NEJMoa1306555 24088041 PMC4211433

[mgg3484-bib-0028] Yang, Y. , Muzny, D. M. , Xia, F. , Niu, Z. , Person, R. , Ding, Y. , … Eng, C. M. (2014). Molecular findings among patients referred for clinical whole‐exome sequencing. JAMA, 312(18), 1870–1879. 10.1001/jama.2014.14601 25326635 PMC4326249

[mgg3484-bib-0029] Yavarna, T. , Al‐Dewik, N. , Al‐Mureikhi, M. , Ali, R. , Al‐Mesaifri, F. , Mahmoud, L. , … Ben‐Omran, T. (2015). High diagnostic yield of clinical exome sequencing in Middle Eastern patients with Mendelian disorders. Human Genetics, 134(9), 967–980. 10.1007/s00439-015-1575-0 26077850

