# Supplementary material for: Clinical whole‐exome sequencing results impact medical management
Source: Mol Genet Genomic Med. 2018 Oct 14;6(6):1068–78. doi: 10.1002/mgg3.484 (PMC6305629; doi:10.1002/mgg3.484)
Supplement: Supplementary file 1 [file MGG3-6-1068-s001.docx]

**Supplemental Figure 1.**

Each person who completes the survey will be entered into a drawing to win a $100 gift card to Amazon.com.

Thank you in advance for your participation! Please take a few moments to provide us with some brief information ***specifically in reference to this patient’s case***. The goal of this research study is to determine how, or if, the results of exome sequencing affect patients’ clinical management. The responses from this survey will be linked to your patient’s information so that we can account for the many variables between different patients’ indications, clinical features, and results. No identifying patient information will be released outside of Ambry Genetics as a result of this study. Neither you nor your patient or patient’s family will be re-contacted in regards to this study.

This is an optional survey consisting of a 19 item checklist with the option to provide additional information, and is expected to take less than 10 minutes. We do not anticipate that taking this survey will impose any significant risk or inconvenience to you. Participation in this study will not likely provide any direct benefits to you or your patient, however our goal is to share the information obtained in this study so that the findings may benefit the care of future patients and families. This study has been IRB approved. If you have any questions regarding IRB approval for this study, please contact Solutions IRB directly at phone number 1-855-226-4472. If you have any questions or comments about the study, please contact Christina Alamillo, MS, CGC at calamillo@ambrygen.com.

**In what ways did the results of exome sequencing affect the management plan for your patient? (check all that apply)**

**MEDICATIONS Primary Result Secondary Result**

Prescription of new medication or supplement □ □

Discontinuation of unnecessary medication or supplement □ □

Discontinuation of medication with potential adverse effects □ □

**TESTING AND REFERRALS**

Referral to additional specialist(s) □ □

Discontinuation of further diagnostic studies □ □

Discontinuation of additional genetic testing □ □

**OTHER MEDICAL MANAGEMENT**

Investigation for additional manifestations of the genetic condition □ □

Availability of enhanced surveillance and/or prophylactic surgery □ □

Change of prognosis expectations □ □

Availability/eligibility of clinical trial □ □

**PSYCHOSOCIAL**

Referral to support group or organization □ □

Availability of additional educational services, social services and/or patient advocacy □ □

Altered the family’s financial planning □ □

**FAMILY PLANNING**

Changed presumed inheritance pattern □ □

Established accurate recurrence risks □ □

Enabled reproductive planning/testing options □ □

**(**e.g. preimplantation genetic diagnosis, prenatal diagnosis, and/or decision to have additional children)

Gained option for carrier testing for family members □ □

**OTHER**

Enabled earlier diagnosis of an affected or pre-symptomatic relative □ □

No significant changes overall □ □

Additional change(s), please specify:

Other comments:
